# Three-dimensional visual technique based on CT lymphography data combined with methylene blue in endoscopic sentinel lymph node biopsy for breast cancer

**DOI:** 10.1186/s40001-022-00909-3

**Published:** 2022-12-04

**Authors:** Baiye Wang, Caifeng Ou, Jingang Yu, Jianping Ye, Yunfeng Luo, Yu Wang, Pusheng Zhang

**Affiliations:** 1 Department of Radiology, Zhujiang Hospital, Southern Medical University, Guangzhou, Guangdong China; 2 Present Address: Department of Breast Care Surgery, The First Affiliated Hospital of Guangdong Pharmaceutical University, Guangzhou, Guangdong 510080 China; 3 Department of Breast Surgery, Zhujiang Hospital, Southern Medical University, 253, Gongye Dadao Zhong, Haizhu District, Guangzhou, 510282 Guangdong China; 4 School of Automation Science and Engineering, South China University of Technology, Guangzhou, Guangdong China; 5 Shenzhen Smart Vision Co. LTD., Shenzhen, Guangdong China; 6 Department of Pathology, Zhujiang Hospital, Southern Medical University, Guangzhou, Guangdong China

**Keywords:** Three-dimensional visualization technique, CT lymphography, Endoscopic sentinel lymph node biopsy

## Abstract

**Background:**

The combined application of blue dye and radioisotopes is currently the primary mapping technique used for sentinel lymph node biopsy (SLNB) in breast cancer patients. However, radiocolloid techniques have not been widely adopted, especially in developing countries, given the strict restrictions on radioactive materials. Consequently, we carried out a retrospective study to evaluate the feasibility and accuracy of three-dimensional visualization technique (3DVT) based on computed tomography-lymphography (CT-LG) in endoscopic sentinel lymph node biopsy (ESLNB) for breast cancer.

**Methods:**

From September 2018 to June 2020, 389 patients who underwent surgical treatment of breast cancer in our department were included in this study. The CT-LG data of these patients were reconstructed into digital 3D models and imported into Smart Vision Works V1.0 to locate the sentinel lymph node (SLN) and for visual simulation surgery. ESLNB and endoscopic axillary lymph node dissection were carried out based on this new technique; the accuracy and clinical value of 3DVT in ESLNB were analyzed.

**Results:**

The reconstructed 3D models clearly displayed all the structures of breast and axilla, which favors the intraoperative detection of SLNs. The identification rate of biopsied SLNs was 100% (389/389). The accuracy, sensitivity, and false-negative rate were 93.83% (365/389), 93.43% (128/137), and 6.57% (9/137), respectively. Upper limb lymphedema occurred in one patient 3 months after surgery during the 12-month follow-up period.

**Conclusions:**

Our 3DVT based on CT-LG data combined with methylene blue in ESLNB ensures a high identification rate of SLNs with low false-negative rates. It, therefore, has the potential to serve as a new method for SLN biopsy in breast cancer cases.

## Background

Worldwide, breast cancer is the most commonly diagnosed malignancy and also the leading cause of cancer-related deaths in women. Approximately 2.3 million new cases of invasive breast cancer occurred globally in 2020, which accounts for almost 1 in 4 cases of cancer among women [1]. As the first lymphatic drainage site of breast cancer, the sentinel lymph node (SLN) represents the status of all axillary lymph nodes (ALNs). Involvement of ALNs is a critical prognostic factor in patients with early-stage breast cancer, as it is valuable both for tumor staging and clinical decision-making [2]. To detect the existence of nodal metastasis, sentinel lymph node biopsy (SLNB) has rapidly emerged and gradually replaced axillary lymph node dissection (ALND) as a standard treatment option for early-stage breast cancer patients with clinically nonsuspicious nodes [3, 4].

In general, the sentinel lymph nodes in breast cancer are located in a small area between the lateral border of the pectoralis major muscle and the lowest edge of the axillary hairline. A lymphatic tracer was injected subcutaneously at 2 to 4 points on the areola during SLNB. An incision was then made near the anatomical location of the SLNs to search for marked or stained lymphatic vessels and SLNs, which was removed for frozen section examination. Currently, intraoperative sentinel node lymphatic mapping for breast cancer is dependent on the injection of tracers, such as blue dye and radioactive colloid [5]. Several studies have established the validity of these tracing reagents with relatively high detection rates, which vary with blue dye (68–86%) and radioisotopes (86–99%) [5–7]. Nevertheless, each of these methods presents several drawbacks. For instance, using blue dye as the sole tracer may result in a low detection rate, and successful detection of SLN is highly dependent on the breast surgeon’s skills. Another notable disadvantage of using blue dye is that the lymphatic drainage pathway and location of SLNs cannot be indicated from the skin surface owing to the dye’s limited penetration [8]. Although using blue dye in combination with radioisotopes is internationally recommended because of its low false-negative rate (7.5%), this practice has not been widely adopted especially in developing countries due to the strict restrictions on radioactive materials. Moreover, this technique has significant limitations such as high cost, radioactive facility requirements, and radiation exposure risk [7, 9]. Thus far, the optimal method of SLN identification remains debatable.

To overcome these deficiencies, we used a new technique: a preoperative three-dimensional visualization technique (3DVT) based on computed tomography-lymphography (CT-LG) data combined with methylene blue in SLNB to accurately locate SLNs. We also evaluated the feasibility and accuracy of this method in patients treated by this method.

## Methods

### Patients

Between September 2018 and June 2020, 389 patients with stage I or II breast cancer received endoscopic sentinel lymph node biopsy (ESLNB) and endoscopic axillary lymph node dissection (EALND) in the Department of Breast Surgery of Zhujiang Hospital. All surgical procedures were performed by the authors of this study. None of the patients received neoadjuvant therapy. After surgery, all patients received standardized treatment in accordance with the National Comprehensive Cancer Network (NCCN) guidelines, including chemotherapy, radiotherapy, and endocrine therapy. The clinical and demographic characteristics of these patients are listed in Table 1.

**Table 1:** Clinical and demographic characteristics of the 389 patients

	*n*	Percentage (%)
Age (years)*	41.8 (22–63)	
BMI (kg/m^2^)*	21.1 (16.5–32.3)	
< 18.5	78	20
18.5–23.9	243	62.5
≥ 24.0	68	17.5
Tumor size (cm)*	2.1 (0.6–3.7)	
Location of the tumor		
Outer upper quadrant	136	35
Outer lower quadrant	107	27.5
Inner upper quadrant	78	20
Inner lower quadrant	68	17.5
Pathologic type		
Invasive ductal carcinoma	282	72.5
Ductal carcinoma in situ	78	20
Pleomorphic carcinoma	10	2.5
Invasive lobular carcinoma	19	5
Tumor stage		
Stage I	168	43.2
Stage II	221	56.8

*Values are presented as median (range)

The patients were selected according to the following criteria:

Inclusion criteria: (1) confirmed diagnosis of stage I or II breast cancer through histologic examination; (2) no suspicious signs of axillary lymph node metastasis were found in physical examination and radiological imaging tests; (3) no distant metastasis.

Exclusion criteria: (1) a history of surgery or radiotherapy to the axillary area; (2) patients with a history of iodine allergy, or hypersensitivity to iopamidol; (3) unsuitable for surgery or refused surgery; (4) pregnant and lactating women; (5) inflammatory breast carcinomas.

### CT lymphography

**Fig. 1:**
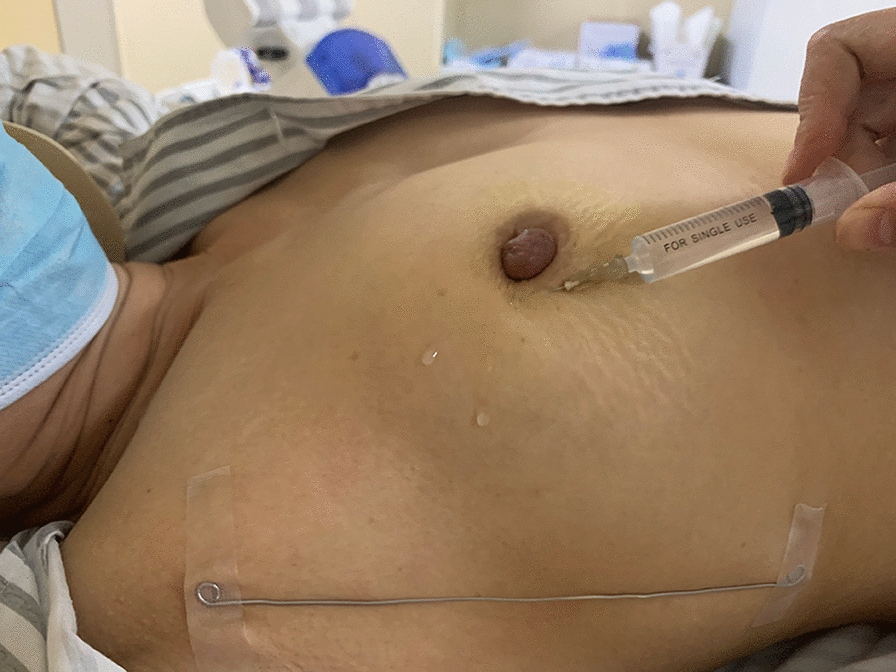
Injection of the contrast agent. 4 mL of lymphatic contrast agent was intradermally injected into the periareolar region in four (clock-wise) quadrants of the breast before CT lymphography

All patients underwent CT-LG before surgery. A total of 4 mL of lymphatic contrast agent (an admixture of 2% lidocaine [3 mL] with 1 mL iopamidol injection [30 g/100 mL], Shanghai Boleco Xinyi Pharmaceutical Co., Ltd.) was administered intradermally at a quarter dose into each quadrant of the areola [10]. After 1 min of mammary massage, 256-multislice CT scanning was performed to collect enhanced CT image data of the SLNs (Fig. 1).

### Image segmentation and digital 3D reconstruction

The CT-LG data of 389 patients were introduced into Smart Vision Works V1.0, software developed by the author, Jianping Ye, for individualized segmentation and 3D reconstruction. The CT data of the axilla and its related structures including the lymph nodes, axillary veins, pectoralis major, pectoralis minor, latissimus dorsi, ribs, and skin were transformed into digital 3D models [11].

### Sentinel lymph node localization

As seen from the 3D images, the contrast agent entered the lymph nodes from the injection site along the lymphatic vessels, and the first enhanced lymph node was considered the SLN. According to the 3D models, on which the relationship among the SLN, pectoralis major, and pectoralis minor is presented, the position of the SLN was marked on the patient’s skin. The imaging results were analyzed and reviewed by a radiologist and a professional breast clinician.

### Visual simulation surgery and clinical surgery

**Fig. 2:**
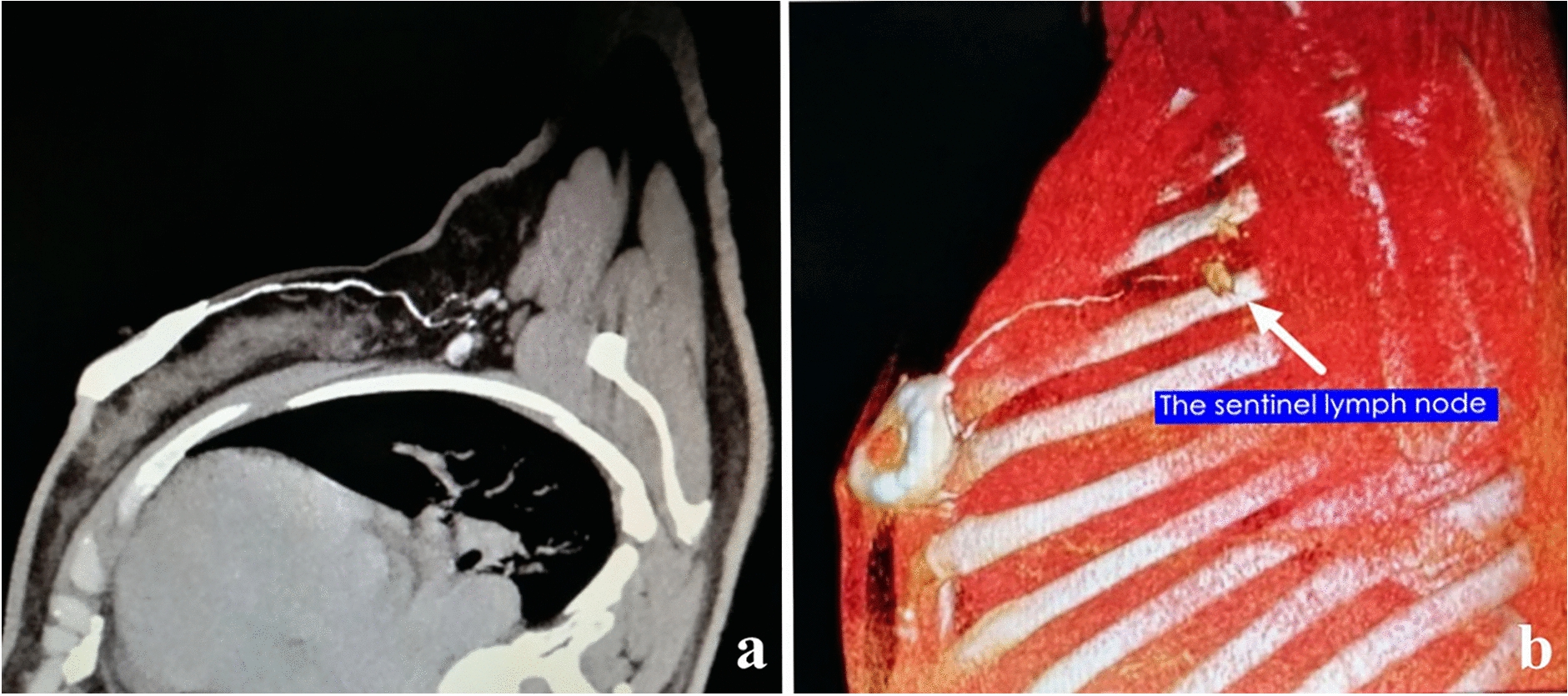
Three-dimensional CT lymphography. **a** CT-LG provided a clear visualization of the lymphatic vessels and sentinel lymph nodes in the axillary region. **b** The sentinel lymph node (white arrow) was located on the reconstructed digital 3D model

The reconstructed 3D models were subsequently imported into Smart Vision Works V1.0 for visual simulation surgery before actual surgery (Fig. 2).

**Fig. 3:**
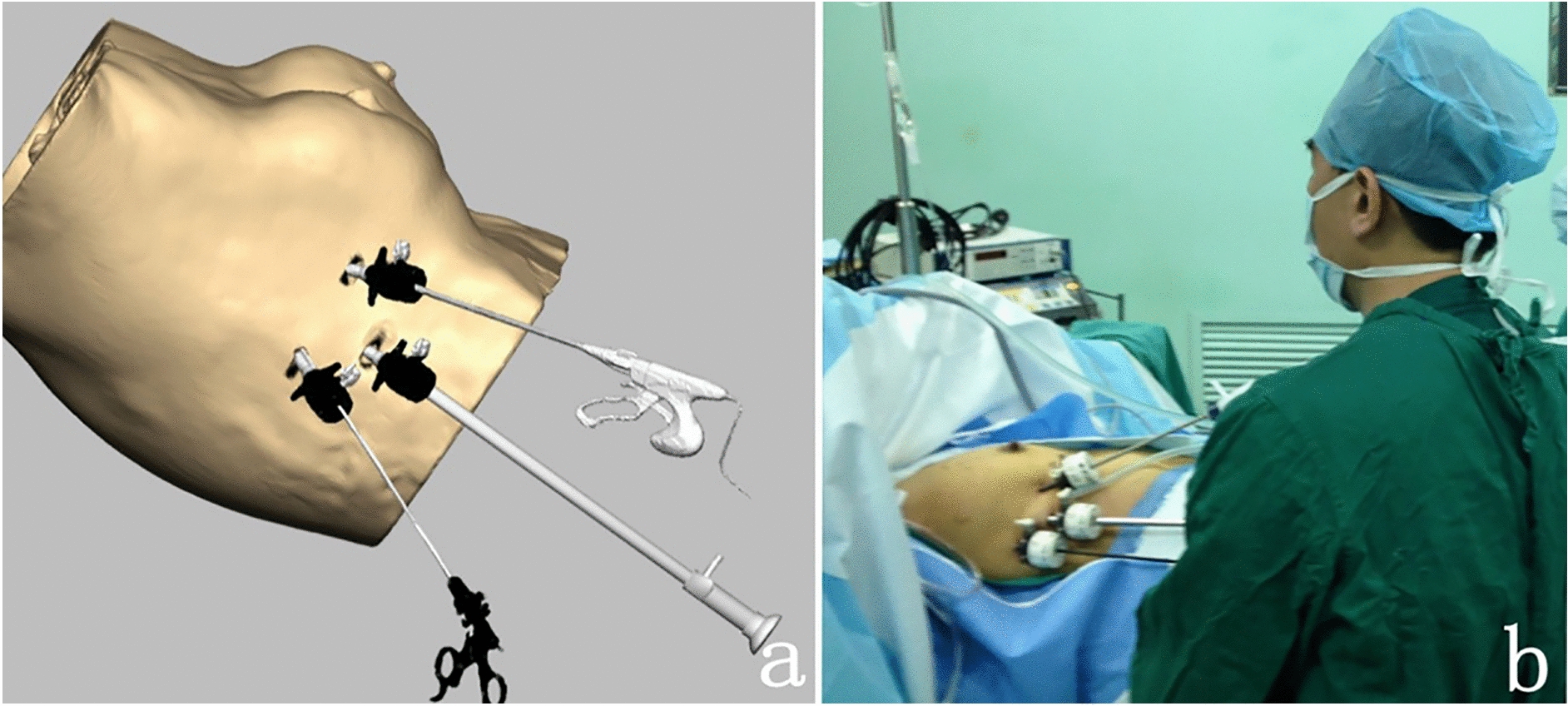
The procedure of visual simulation surgery and clinical surgery. **a** Ultrasound scissors and forceps were placed, and the lymph nodes were dissected in the visual simulation surgery. **b** Endoscopic sentinel lymph node biopsy and axillary lymph node dissection in the actual surgery

The details of this process are as follows:

1. 0.5 mL of methylene blue (2 mL: 20 mg, JUMPCAN Pharmaceutical Group Co., Ltd.) was intradermally injected into the periareolar region in four (clock-wise) quadrants of the breast, 10 min before surgery.
2. A 10-mm transverse incision was made along the mid-axillary line at the level of the lower margin of the breast as an inspection incision using a simulated scalpel. Two additional 5-mm operating incisions were made in the anterior axillary line at the nipple level (the first incision) and the posterior axillary line at the nipple level (the second).
3. The channel was expanded with a balloon, followed by insertion of a 10-mm trocar into the inspection hole. CO_2_ was infused to maintain pressure at 11 mmHg (1 mmHg = 0.133 kPa). Then, a 30° endoscope was placed into the trocar.
4. Two 5-mm trocars were placed in the first and second operating incision, respectively, and ultrasonic scissors and grasping forceps were inserted to perform ESLNB and EALND (Fig. 3).
5. We looked for the blue-stained lymphatic vessels and SLN along the intersection of the lateral margin of the pectoralis major and the lower edge of the mane according to the mark presented of the SLN on the body surface, and then removed 2–5 SLNs for rapid frozen pathological examination.
6. After thorough hemostasis, the axilla was cleaned with distilled water, and a rubber drain tube was placed through the inspection incision to avoid fluid accumulation.
7. Finally, the skin incision was closed with a simulated suture.

The clinical surgery was carried out according to the procedure of the visual simulation surgery based on the 3DVT. In ESLNB, 2–5 SLNs of each patient were removed intraoperatively. If there were no positive nodes, only the level I lymph nodes were removed. Otherwise, the axillary level II lymph nodes were dissected, which occasionally accompanied level III nodes when enlarged lymph nodes were detected. All resected ALNs were sent for pathological examination. We compared and analyzed the pathological results of the SLNs and ALNs after surgery. Furthermore, the identification rate, accuracy, sensitivity, and false-negative rate of SLN were calculated to evaluate the application value of CT-LG with 3DVT in ESLNB.

Follow-up physical examination was performed every 3 months after the surgery. We evaluated the incidence of postoperative complications by measuring the circumference of the upper arm, examining the sensation and mobility of the arm, and checking the surgical incisions for infection and the presence of axillary effusion in the axilla.

## Results

### Individualized digital 3D reconstruction results

**Fig. 4:**
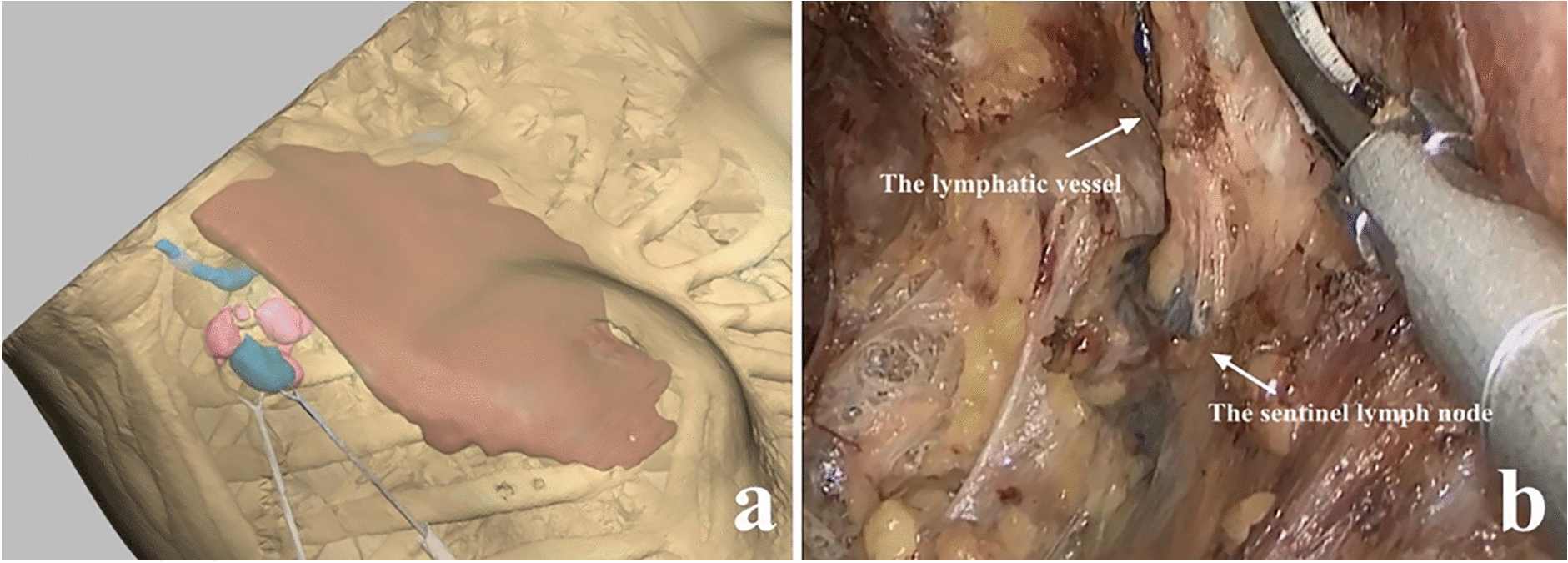
Sentinel lymph node localization in visual simulation surgery and clinical surgery. **a** Sentinel lymph node was located accurately, and endoscopic sentinel lymph node biopsy and axillary lymph node dissection were performed smoothly in the simulation surgery. **b** Blue-stained sentinel lymph nodes and axillary lymph nodes were resected under endoscopy in the actual surgery

The 3D models reconstruction fully represented the surgical anatomy of the patients. Because the 3D reconstruction model could be amplified, hyalinized, and rotated at multiple angles, the relationship among the SNLs, ALNs, axillary vein, pectoralis major, pectoralis minor muscle, and latissimus dorsi were clearly displayed (Fig. 4).

### Intraoperative and postoperative results

According to the 3DVT procedure based on CT-LG data combined with methylene blue, clinical surgery was carried out and the SLN was accurately detected during the surgery. We resected 1323 SLNs from 389 patients in total, with an average of 3.4 per patient. Among these, 36, 181, 152, and 20 patients had two, three, four, and five SLNs removed, respectively. All 389 patients underwent EALND after ESLNB, during which a total of 4278 ALNs, including level I, II, and some level III lymph nodes were dissected. The results of pathological examination revealed that 128 cases had positive SLNs and ALNs, while 237 cases had negative SLNs and ALNs. Nine patients had negative SLNs but positive ALNs, while 15 patients had positive SLNs and negative ALNs.

Considering the pathological results of ALNs as a control standard, the identification rate of ESLNB was 100% (389/389) in this group with an accuracy of 93.83% (365/389), sensitivity of 93.43% (128/137), and false-negative rate of 6.57% (9/137) (Table 2).

**Table 2:** Results of ESLNB and EALND

ESLNB	EALND		
	Positive	Negative	Total
Positive	128	15	143
Negative	9	237	246
Total	137	252	389
	Sensitivity = 93.43% (128/137)	Accuracy = 93.83% (365/389)	
	False-negative rate = 6.57% (9/137)	Identification rate = 100% (389/389)	

*ESLNB* endoscopic sentinel lymph node biopsy, *EALND* endoscopic axillary lymph node dissection

Upper limb lymphedema occurred in one patient 3 months after surgery. No other complications such as limb pain, swelling, wound infection, and subcutaneous edema were found in any patients during the 12-month follow-up period.

## Discussion

In this study, we found that our reconstructed 3D models clearly displayed all the structures of breast and axilla, which favors the intraoperative detection of SLNs. Our data demonstrated that the method of ESLNB guided by preoperative 3DVT and intraoperative methylene blue dye exhibited a high SLN identification rate of 100% (389/389), sensitivity of 93.43% (128/137) and an accuracy of 93.83% (365/389), with a relatively low false-negative rate of 6.57% (9/137). Surgical complications of upper limb lymphedema occurred in one patient during the 12-month follow-up period after surgery.

A comprehensive understanding of lymphatic drainage pathways and precise determination of the location of SLNs through preoperative examinations are critical to the success of SLNB. In this study, we applied a 3DVT based on CT-LG data combined with methylene blue to identify the SLNs, and 3D reconstruction with visual simulation surgery were conducted as routine procedures before actual surgery. With this technique, traditional two-dimensional CT data can be reconstructed into a 3D visualization model, which allows for a clear visualization of the breast structure, the spatial location of lymph nodes, and their relationship with surrounding tissues. Thus, surgeons could locate the SLNs and design surgical plans accordingly, and the position of the endoscopic incision and direction of the long syringe could also be easily determined [12]. Therefore, we consider this 3D visualization technique to offer efficient detection and accurate removal of SLNs intraoperatively.

Studies have revealed the superiority of the combined use of blue dye and radioisotopes in terms of relatively low non-identification and false-negative rates. Hence, this dual technique is currently considered the international standard method and has been recommended for SLN mapping [13, 14]. However, the radioisotope method is not extensively accessible in clinical practice. Most of medical institutions in developing countries such as China still mapping breast cancer SLNs using blue dye alone. Using blue dye as the sole tracer inevitably leads to a relatively low detection rate (89% to 95.7%) and high false-negative rate (9% to 18%), as reported in previous studies [6, 8, 15]. In terms of diagnostic accuracy of SLN, our result was similar to or even better than those of previous studies that identified SLN by indocyanine green or a conventional dual method [7, 16]. Thus, it can be said that blue dye guided by 3DVT is sufficient for SLN detection in breast cancer.

With the navigation of 3DVT and preoperative CT-LG, the present study showed a false-negative rate of 6.57% (9/137). Compared to the false-negative rates reported in other studies performing SLNB or ESLNB without the assistance of 3DVT and preoperative CT-LG, our results showed a modest reduction in the false-negative rate, which further reinforces the advantages of preoperative 3D CT-LG and visual simulation surgery [17, 18]. The reasons for the satisfactory SLN detection performance of this technique in our study are as follows. The digital 3D model reconstructed from CT-LG data is virtually identical to the patient's anatomy. These models help to determine the number and location of lymph nodes in advance, which facilitates the design of surgical plans. Due to the visualized simulation surgical procedure based on the digital 3D models, we can quickly and accurately find SLNs during surgery. This method simplifies the process of searching for SLNs and reduces the probability of omission. Therefore, applying 3DVT based on preoperative CT-LG in ESLNB results in the ability to detect and identify SLNs with a high identification rate and a relatively low false-negative rate.

In this group of patients, nine cases of false-negative results occurred with one or two positive ALNs after the final dissection. Related factors that were associated with failure of SLN identification included body mass index, size or location of the tumor, navigation technique, and surgeon experience. The last factor might explain our failure to harvest the SLNs, as all of these cases occurred during the early stage of our study. Positive SLNs might have been omitted because of insufficient experience in SLN localization based on CT-LG and intraoperative use of methylene blue dye. As the surgeons became more proficient with the procedure, no false-negative results were identified in the patients enrolled later.

During the 12-month follow-up period, one patient developed lymphedema of the upper limb 3 months after the surgery; the patient’s arm was swollen and she found it difficult to move. Complications such as limb pain, swelling, wound infection, and subcutaneous edema were not found in other patients during the 12-month follow-up period. The possible reasons for the lower incidence of lymphedema are as follows. The 3D model reconstructed based on CT-LG data allows for a clear visualization of the spatial location of lymph nodes and their relationship to the surrounding tissue. The application of 3DVT in ESLNB enables accurate detection and removal of SLNs, and helps avoid damage to additional tissues and structures in the armpit. In addition, as a less invasive procedure, ESLNB provides excellent cosmetic results by reducing the size of the operative incision and minimizing tissue trauma, which is consistent with the esthetic needs of female patients, especially those that require breast conservation or reconstruction [19, 20]. By using an endoscope, the surgical field of view is much clearer to preserve the small veins and lymphatic vessels that drain the upper limb. In this way, the occurrence of postoperative lymphedema can be minimized greatly.

Our research suggests that 3DVT enables surgeons to have a more accurate understanding of the patient’s internal anatomical structure preoperatively, which is conducive to reasonable surgical planning [21, 22]. It also plays a guiding role during the actual surgery, assisting surgeons to quickly locate SLNs and avoid damaging additional tissue, thereby reducing complications.

This study has some limitations. First, the relatively small sample size from a single institution might compromise the representativeness of the study. Second, short follow-up time and the lack of long-term efficacy tracking data do not allow further assessment of oncological outcomes. Therefore, more high quality, large-scale randomized controlled clinical trials are needed to verify the current findings.

## Conclusions

The 3DVT based on preoperative CT-LG data combined with intraoperative methylene blue dye in ESLNB detected and identified SLNs with high sensitivity and a relatively low false-negative rate. This method provides high feasibility owing to its low invasiveness and short operating time, thereby reducing patients’ distress and improving quality of life. This technique provides a safe and practical approach to evaluate the status of ALNs. Consequently, it could be used as a promising alternative to other established SLN mapping modalities to benefit more breast cancer patients.

## Data Availability

The datasets generated and analyzed during the current study are not publicly available due to individual privacy, data protection and confidentiality, but are available from the corresponding author on reasonable request.

## Abbreviations

3DVT: Three-dimensional visualization technique
SLNB: Sentinel lymph node biopsy
SLN: Sentinel lymph node
ALN: Axillary lymph node
ALND: Axillary lymph node dissection
ESLNB: Endoscopic sentinel lymph node biopsy
EALND: Endoscopic axillary lymph node dissection
CT-LG: Computed tomography-lymphography
