# Correction: Three-dimensional visual technique based on CT lymphography data combined with methylene blue in endoscopic sentinel lymph node biopsy for breast cancer

**DOI:** 10.1186/s40001-024-02123-9

**Published:** 2024-11-05

**Authors:** Baiye Wang, Caifeng Ou, Jingang Yu, Jianping Ye, Yunfeng Luo, Yu Wang, Pusheng Zhang

**Affiliations:** 1 Department of Radiology, Zhujiang Hospital, Southern Medical University, Guangzhou, Guangdong China; 2 Present Address: Department of Breast Care Surgery, The First Affiliated Hospital of Guangdong Pharmaceutical University, Guangzhou, 510080 Guangdong China; 3 Department of Breast Surgery, Zhujiang Hospital, Southern Medical University, 253, Gongye Dadao Zhong, Haizhu District, Guangzhou, 510282 Guangdong China; 4 School of Automation Science and Engineering, South China University of Technology, Guangzhou, Guangdong China; 5 Shenzhen Smart Vision Co. LTD., Shenzhen, Guangdong China; 6 Department of Pathology, Zhujiang Hospital, Southern Medical University, Guangzhou, Guangdong China

**Correction: European Journal of Medical Research (2024) 27:274** 10.1186/s40001-022-00909-3

In the original article [1], the image of the patient in Fig. 1 has the patient’s face visible. It requires explicit informed consent for identifiable details or images of research participants to be published and not satisfied that this has been met for this case as the eye bar provides sufficient anonymisation to the patient.

The authors have provided revised image (Fig. 1).

**Fig. 1:**
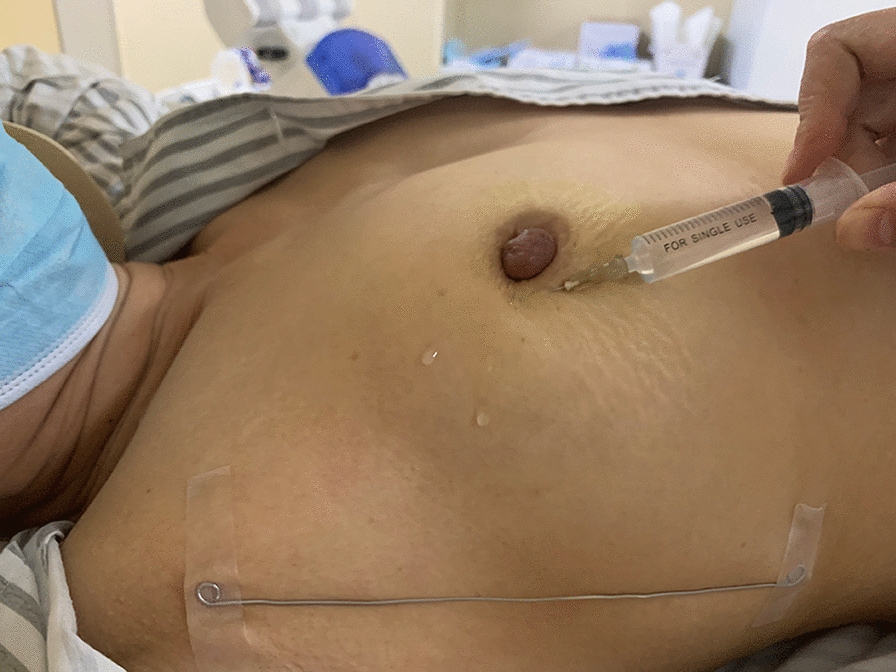
Injection of the contrast agent. 4 mL of lymphatic contrast agent was intradermally injected into the periareolar region in four (clock-wise) quadrants of the breast before CT lymphography

The original article has been corrected.
